# Bacteriorhodopsin-Based pH Sensor for Cell Culture Condition Regulation

**DOI:** 10.3390/ma18030478

**Published:** 2025-01-21

**Authors:** Jiayin Huang, Shiwang Xie, Haoqi Fan, Chen Song, Qiang Zheng, Dan Luo, Zhu Zeng, Zhou Li, Yujia Lv

**Affiliations:** 1 Key Laboratory of Infectious Immune and Antibody Engineering of Guizhou Province, Engineering Research Center of Cellular Immunotherapy of Guizhou Province, School of Biology and Engineering, Guizhou Medical University, Guiyang 561113, China; huangjiayin@binn.cas.cn; 2 Beijing Institute of Nanoenergy and Nanosystems, Chinese Academy of Sciences, Beijing 101400, China; xieshiwang@binn.cas.cn (S.X.); fanhaoqi@binn.cas.cn (H.F.); songchen@binn.cas.cn (C.S.); 3 Immune Cells and Antibody Engineering Research Center of Guizhou Province, Key Laboratory of Biology and Medical Engineering, Guizhou Medical University, Guiyang 561113, China; 4 Engineering Research Center of Intelligent Materials and Advanced Medical Devices, School of Biology and Engineering, Guizhou Medical University, Guiyang 561113, China; 5 School of Nanoscience and Technology, University of Chinese Academy of Sciences, Beijing 100049, China; 6 Key Laboratory of Urban Stormwater System and Water Environment, Ministry of Education, Beijing University of Civil Engineering and Architecture, Beijing 100044, China; 7 Faculty of Electronic Information and Automation, Tianjin University of Science and Technology, Tianjin 300457, China

**Keywords:** bacteriorhodopsin, pH sensor, cell culture

## Abstract

In cell culture research and biotechnology, precise pH monitoring is crucial for maintaining cellular health and ensuring reliable experimental outcomes. Traditional pH measurement methods, such as glass electrodes and chemical indicators, are often limited by issues such as fragility, calibration requirements, and potential cytotoxicity. This study presents a novel pH sensor based on bacteriorhodopsin (bR), a light-sensitive protein that undergoes conformational changes in response to pH fluctuations, generating a measurable photoelectric signal. The integrated bR-based electrochemical electrode in a flexible pH biosensor is demonstrated, with measurements spanning the physiological pH range of 6.0–8.5. The sensor shows a high correlation (R^2^ = 0.977) between photo-generated current signals and pH, indicating robust performance for real-time, non-invasive pH monitoring. The biocompatibility and non-invasive nature of this sensor make it particularly suitable for continuous monitoring in cell culture environments. The sensor’s practical application is validated by its integration into cell well plates for tracking the pH changes during cell growth, providing valuable insights into metabolic processes and growth conditions. In the future, efforts will focus on enhancing sensor sensitivity, stability, and integration with multi-parameter monitoring systems for more comprehensive cell culture analysis.

## 1. Introduction

In modern cell culture research and biotechnology applications, precise control and monitoring of the culture environment are essential for obtaining reliable and reproducible results. The pH, as a fundamental parameter, plays a critical role in cell growth, metabolism, protein synthesis, and overall cell viability [1]. Slight deviations from the optimal pH range can lead to significant alterations in cell behavior and function, potentially compromising the success of cell culture experiments and bioprocesses [2].

Common pH sensors include glass electrode pH sensors, ion-selective electrode (ISE) pH sensors, and semiconductor-based pH sensors. Traditional pH measurement methods in cell culture often involve the use of glass electrodes or chemical indicators. While these approaches have been widely used, they possess certain limitations [3]. Glass electrodes can be bulky, fragile, and may require frequent calibration. Chemical indicators, on the other hand, provide only a qualitative or semi-quantitative assessment and may introduce additional chemicals into the culture system, which could potentially affect cell health [4]. There is an urgent need for a pH detection method that is both highly accurate and easily integrated into existing protocols. This method should also minimize contamination caused by contact, which is a major concern in cell culturing.

Bacteriorhodopsin (bR), a light-sensitive transmembrane protein found in certain microorganisms, has recently attracted considerable attention as a potential candidate for pH sensing [5]. The unique photochemical properties of bR, particularly its ability to undergo conformational changes and proton translocation in response to light, offer an innovative approach to pH measurement [6]. The relationship of the forward-to-reverse current ratio versus pH was found to be linear within the pH range of pH 3.5–9, which could enable utilizing bR as a pH sensor [7]. When illuminated, bR initiates a photocycle that involves the transfer of protons across the membrane, generating a measurable photoelectric signal that is correlated with the surrounding pH [8].

Nevertheless, the progress and utilization of a pH sensor in the context of cell culture condition modulation are accompanied by several obstacles. One of the primary concerns is the optimization of sensor sensitivity and selectivity [9]. Ensuring that the sensor can accurately detect small pH variations within the physiologically relevant range and distinguish between pH changes and other potential interfering factors is essential for reliable data acquisition [10]. Additionally, the long-term stability of the sensor, both in terms of its physical integrity and its sensing performance, needs to be enhanced. Exposure to the complex and often harsh cell culture environment, including temperature fluctuations, nutrient gradients, and the presence of various metabolites, can potentially affect the sensor’s functionality over time [11].

The integration of a bR-based pH sensor in cell culture systems holds several significant advantages. Firstly, bR is a natural biomolecule and is less likely to cause cytotoxicity or adverse effects on cultured cells compared to some traditional sensor materials. This makes it an ideal candidate for long-term and in situ pH monitoring in cell cultures [12]. Secondly, optical sensing presents remarkable advantages. It showcases rapid responsiveness, empowering it to promptly detect and record even the slightest alterations. Moreover, it enables non-contact measurement, a feature that is highly valuable. This non-invasive property permits seamless, continuous, and real-time pH value monitoring without causing any perturbations to the cell culture environment. Given that the stability of the cell culture environment is of utmost importance, any interference could introduce bias to experimental results or inflict damage upon the cells, undermining the entire study. This is crucial for capturing dynamic pH changes that occur during cell growth and various physiological processes [13]. Furthermore, the electrodes used in optical sensing are both small in size and lightweight. These attributes make them highly amenable to integration into a wide array of micro- and nano-devices, as well as lab-on-a-chip systems. Such seamless integration paves the way for the development of more advanced, miniaturized analytical instruments in scientific research.

Within this thesis, an integrative electrochemical electrode founded on the photosensitive protein biomaterial bR is introduced. This electrode is applied in a pH-based biomaterials flexible wearable pH biometer. The preparation and characterization of the photosensitive protein bR photoelectrode are described. Measurements were carried out within the pH range from 6.0 to 8.5, wherein a favorable linear correlation was observed between the photo-generated voltage response signals (the ratio of positive photocurrent (Ip) to negative dark current (In)) and the pH (R^2^ = 0.977). The practical application potential of the pH biosensor for pH monitoring was further validated by integrating the bR-based pH sensor into cell well plates to track the pH alterations during cell growth. The non-invasive pH monitoring approach not only exhibits biocompatibility but also enables the monitoring of the cell growth cycle, determination of the medium change time, and modulation of cell growth conditions.

## 2. Materials and Methods

### 2.1. Materials

NaCl, NaOH, KCl, Na_2_HPO_4_, and KH_2_PO_4_ (pH 6.0, 6.5, 7.0, 7.5, 8.0) were purchased from Aladdin, Shanghai, China. The PET film was purchased from Shenzhen Lelin technology development Co., Ltd., Shenzhen, China. The platinum, carbon, and silver/silver chloride were purchased from Meryer Chemical Technology Co., Ltd., Shanghai, China. Calcein-AM (C2015M-Beyotime) was purchased from Beijing Suolaibao technology Co., Ltd., Beijing, China. These chemical medicines used were of analytical grade and no further purification was performed in this study.

### 2.2. Fabrication of bR-Based pH Sensor

The working, counter, and reference electrodes were fabricated through a screen-printing technique on a polyethylene terephthalate (PET) film substrate. Homogeneous conductive inks with suitable viscosities, namely platinum (Pt), carbon, and silver/silver chloride (Ag/AgCl), were utilized for this purpose. For the preparation of the Ag/AgCl reference electrode, a precisely measured volume of 3 µL of Ag/AgCl ink was carefully dispensed onto the designated reference electrode area. Subsequently, the ink was allowed to dry overnight under controlled conditions. The Pt and carbon electrodes were fabricated following an identical procedure to ensure consistency and reproducibility [14].

To prevent short-circuiting during electrochemical testing, a PET insulating layer was applied to the regions of the substrate that were not part of the electrode reactive areas. This insulation layer served as a crucial safeguard, ensuring the integrity of the electrochemical measurements. The pH sensor was constructed by depositing a droplet of the bR suspension (1 mg/mL) onto the surface of the working electrode, which was made of Pt. The water content within the bR suspension was then removed by evaporation at 45 °C until complete dryness was achieved, thereby immobilizing the bR onto the working electrode and forming the functional pH sensor [15]. Finally, deionized washing was used to remove poorly deposited proteins from the film.

### 2.3. Characterization of bR-Based pH Sensor

The SU8020 cold-field scanning electron microscope was purchased from HITACHI Ltd., Tokyo, Japan. The PerkinElmer Lambda 35 UV/vis spectrophotometer was purchased from PerkinElmer Co., Ltd., Shanghai, China. The SU8020 cold-field scanning electron microscope was employed to analyze the surface topography of the bR electrode (operating voltage 5.0 kV, vacuum levels 10^−7^ Pa). The measurement of the UV–vis absorption spectrum was carried out by means of a PerkinElmer Lambda 35 UV/vis spectrophotometer (wavelength range 260–750 nm, wavelength accuracy ±0.1 nm).

### 2.4. Detection of the Photo Signals

The Chi760e electrochemical workstation was purchased from Shanghaichenhua Co., Ltd., Shanghai, China. The xenon lamp (CEL-HXF300) was purchased from Beijing China educational Au-light technology Co., Ltd, Beijing, China. The open-circuit voltage was examined with the Chi760e electrochemical workstation. The three electrodes in the bR-based pH sensor were immersed in an electrolyte, and the conductive electrodes were connected to the electrochemical workstation [16]. The xenon lamp (CEL-HXF300) furnished a continuous broadband light source, possessing a luminous output of 200.0 mW cm^−2^. Control of the light frequency was by means of a cut light plate. The photocurrent and dark current were then measured by the IT model in the electrochemical workstation [17].

### 2.5. Cell Culture

Mouse skin melanoma cell B16-F10 (B16F10) was cultured in Roswell Park Memorial Institute 1640 culture medium (RMPI-1640) containing 10% fetal calf serum and 1% penicillin/streptomycin on a cell dish or 24-well TC-treated multi-well plates in an incubator (37 °C, 5% CO_2_) and grown until they reached 80–90% confluency. The cells were passaged using 0.25% trypsin/EDTA solution and seeded at 2 × 10^5^ cells mL^−1^. For long-term storage, the cells were cryopreserved in liquid nitrogen. The cryopreservation process included harvesting the cells at high density (approximately 90% confluence) that were resuspended in a freezing medium containing 5–10% dimethyl sulfoxide (DMSO) as a cryoprotectant and then transferred to liquid nitrogen for long-term storage within the cryovials [18].

B16-F10 were cultured in the RMPI-1640 medium before the cytotoxicity tests. The bR-based pH sensor was soaked in 20 mL of RMPI-1640 for 72 h, and then the medium was diluted several times in sequence. After PBS washing, trypsin digestion, centrifugation, RMPI-1640 resuspension, and cell counting, B16-F10 was sequentially seeded into 24-well plates and cultured at 37 °C with 5% CO_2_ for 24 and 48 h. The density of the seeded plates was 10^4^ and 6.7 × 10^3^ cells per 100 µL of culture medium. Then, the culture medium was washed with PBS twice, and 110 µL of the cck8 mixture (10 µL of cck8 and 100 µL of the culture medium) was added. After 1–4 h of incubation, A450 was obtained using an enzymatic assay and the results were calculated and analyzed. 

B16-F10 were cultured in a normal attached-wall culture with bR-based pH sensor leachate used for the medium replacement, and they were then placed at 37 °C with 5% CO_2_ for 24 and 48 h. The medium was then washed with PBS and stained with Calcein-AM (C2015M-Beyotime). After the staining treatment was performed, the medium was observed under a microscope and photographed for recording.

### 2.6. The pH Results of Medium

The bR-based pH sensor was integrated into the bottom of the cell culture medium. At the 24th, 36th, 48th, and 72nd hours of cell culture, the photocurrent Ip and In were obtained by turning the light source on and off. By substituting the calculated Ip/In into Equation 1, the pH of the culture medium could be obtained.

## 3. Results

### 3.1. Characterization of the bR-Based pH Sensor

**Figure 1:**
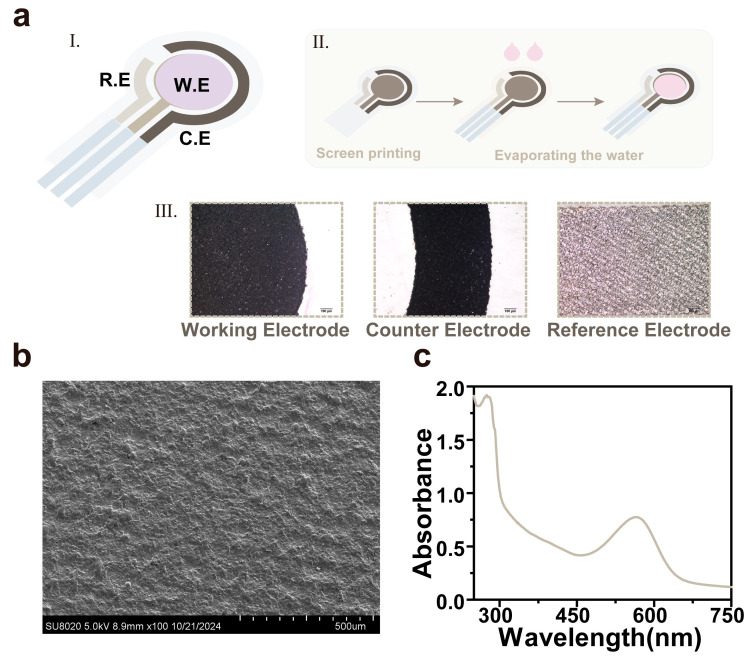
Preparation of the bR-based pH sensor: (**a**) the images of the bR-based pH sensor (I the schematic image of pH sensor, II the preparatory process of pH sensor, III the electrodes pictures in microscopes (scale bar: 100 μm)); (**b**) the SEM image of the bR film; and (**c**) the absorption spectrum of the bR film in the range of 250–750 nm.

Figure 1a shows the schematic diagram of our proposed bR-based pH sensor, which is an integrated three-electrode electrochemical electrode with bR/Pt as the working electrode, carbon as the counter electrode, and Ag/AgCl as the reference electrode. To investigate the physical properties of the bR electrode, we used various methods to characterize it. Basic scanning electron microscopy (SEM) characterized the surface of the film and determined that the Pt electrode was covered with a uniform and dense bR film (Figure 1b). Figure 1c shows the characteristic peaks of bR at both 280 nm and 568 nm, and the ratio of bR suspension A280 to A568 is 2.2. The results indicate that the purity of bR in the solution is relatively high, and there is a low concentration of impurity proteins or other interfering components. Conversely, the presence of a substantial number of impurity proteins would lead to abnormal amino acid residue positions and retinal chromophore configurations within the bR molecule, resulting in significant conformational distortion [19].

### 3.2. The Mechanism of the bR-Based pH Sensor

**Figure 2:**
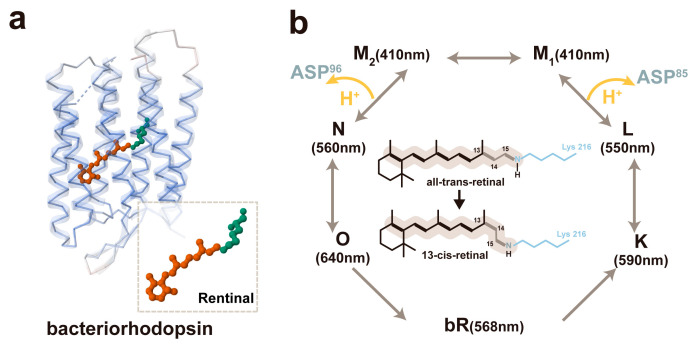
The working mechanism of the bR-based pH sensor: (**a**) the protein structure of bR. (PDB code: 1BRX) and (**b**) the photocycle process of bR.

The bR molecule is a polypeptide strand composed of 248 amino acid residues, which assemble into seven α transmembrane helical bundles on the inner aspect of the lipid membrane (Figure 2a). This molecule also incorporates the chromophore-retinal, which is covalently attached to lysine-216 through a Schiff base [20]. The retinal molecule exists in two isomeric forms, namely all-*trans* and 13-*cis*. In dark-adapted circumstances, it is in the all-*trans* configuration, while in light-adapted conditions, it adopts the 13-*cis* form, with the isomerization of the retinal molecule transitioning from all-trans to 13-*cis*. Under alkaline circumstances, bR becomes photoexcited and experiences a photoperiod of bR-K -L-M-N-O-bR. During the transformation from the L state to the M state, due to coupling, the Schiff base donates a proton, leading to the release of the proton to Asp-85, and the proton is converted to Asp-85-H. The proton is also transported to the exterior of the cell membrane, resulting in a significant reduction in the pKa value of the proton release motif (from pK_a_ = 13.3 to pK_a_ = 2.2) [21]. When Asp-96-H shifts from the M state to the N state, Asp-96-H is deprotonated to Asp-96, and the proton is transferred back to the Schiff base, causing it to be protonated once more. At the transition from the N state to the O state, Asp-96 is reprotonated by absorbing protons from the intracellular compartment, and Asp-85-H is also reprotonated to supply protons to the proton release group, ultimately allowing bR to revert to its original state (Figure 2b). Consequently, when the bR-based pH sensor is illuminated, with darkness serving as the background, and the proton pump is activated instantaneously upon illumination, the rapid conversion of retinal from all-trans to 13-cis and the rapid polarization of the electron cloud in the retina leads to charge separation. Under alkaline conditions, the proton transfer from the Schiff base to the proton acceptor Asp85 (where the proton release groups are expelled from the cell) occurs more rapidly than the proton donation from Asp96 to the Schiff base (where Asp96 imports a proton from the exterior of the cell), thereby resulting in the net pumping of protons from the interior to the exterior of the cell [6].

### 3.3. The Photo Signals of the pH Sensor

**Figure 3:**
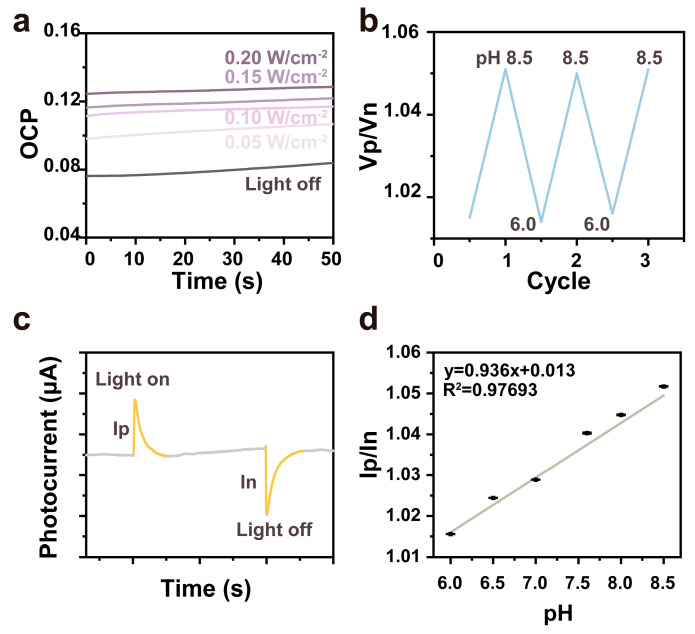
The performance of the bR-based pH sensor: (**a**) the OCP at different light intensities; (**b**) pH for the reversibility of the bR photoelectrode (pH range 6.0-8.5); (**c**) photocurrent and dark current at diverse pH values; and (**d**) the photocurrent and dark current measurements were conducted at various pH levels (n = 6) for the bR-based pH sensor.

The variations in open-circuit voltage (OCP) with light intensity have been demonstrated to reflect the charge transfer processes and kinetic characteristics on the surface of the photoelectrode. In the context of sensors based on the photoelectric effect, such as optical sensors and biosensors, the process, illustrated in Figure 3a as the light-switching process, facilitates an increase in the open-circuit voltage concomitant with the augmentation of light intensity. This, in turn, enables the sensor to respond with heightened sensitivity to optical signals. Under light-adapted conditions, positive signal peaks were detected, whereas negative signal peaks emerged under dark-adapted conditions. During the photocycle, the heterodimerization of the chromophore-retinal within bR gives rise to a charge transfer, thereby generating a potential difference. At different pH values, by inducing the light and dark voltage inversion of the bR electrode in Figure 3b, the rate constants associated with proton release and uptake at the membrane surface are highly contingent upon pH. When the bR electrode is subjected to light exposure, the pH of the solution impacts a sequence of deprotonation and reprotonation processes of the Schiff base and its neighboring amino acids, modifying the normal interactions among them and consequently influencing photocurrent generation. M_1_ undergoes reprotonation via Asp-85 and exhibits a strong dependence on extracellular pH. Subsequently, bR electrodes were evaluated electrochemically, and their photovoltaic characteristics are presented in Figure 3c. Upon illumination, a positive photoresponse signal (Ip) was promptly generated. When transitioning to the dark-adapted state, the cis and trans equilibrium of the retina shifts towards the predominance of the all-trans configuration. The continuous transmembrane conduction current produced by the proton pump, in conjunction with the coupling properties of the measurement circuit, leads to the formation of a potential difference, resulting in the appearance of a negative signal peak, which is labeled In. Leveraging this property, electrochemical analyses were conducted using a biohybrid device to assess the performance of a pH sensor based on an electrochemical electrode. By inducing bright and dark voltage inversions of the bR electrode at diverse pH values, an outstanding linear relationship between Ip/In and pH within the range of 6.0–8.5 (R^2^ = 0.97693, n = 6) was observed [22].

Equation (1) defines a quantitative relationship between Ip/In and pH (Figure 3d).

y = 0.936x + 0.013(1)

Linear regression is a commonly employed statistical analysis technique for ascertaining quantitative associations between distinct variables within a dataset and for exploring the correlation between variables. The independent and dependent variables can be mutually derived based on the established quantitative relationships. Moreover, the obtained regression equations can be validated through statistical tests, and their ability to mirror the actual scenario and possess practical significance can be appraised in accordance with the significance level (α), which is related to the sample size (n) and the correlation coefficient (R^2^).

### 3.4. Medium pH Monitoring with pH Sensor

To make the bR-based pH sensor suitable for metabolic monitoring in cell culture, several requirements must be met. Firstly, integrated electrochemical electrodes are essential to meet the demands of the culture environment. Secondly, the device needs to be sterilizable prior to use. Thirdly, it must possess biocompatibility with cultured cells [23]. Concerning the biocompatibility aspect, silver ions or silver nanoparticles from traditional Ag/AgCl reference electrodes can interact with metabolic enzymes and nucleic acids. This interaction may disrupt the cellular functions of biomolecules and has cytotoxic potential. Moreover, commercial Ag/AgCl electrodes do not integrate well into cell culture well plates. Compared to conventional silver chloride, screen-printing technology allows for more precise control of the thickness and structure of the silver chloride layer, thus reducing the release of silver ions to a certain extent [24] (refer to Supplementary Materials Figure S1).

**Figure 4:**
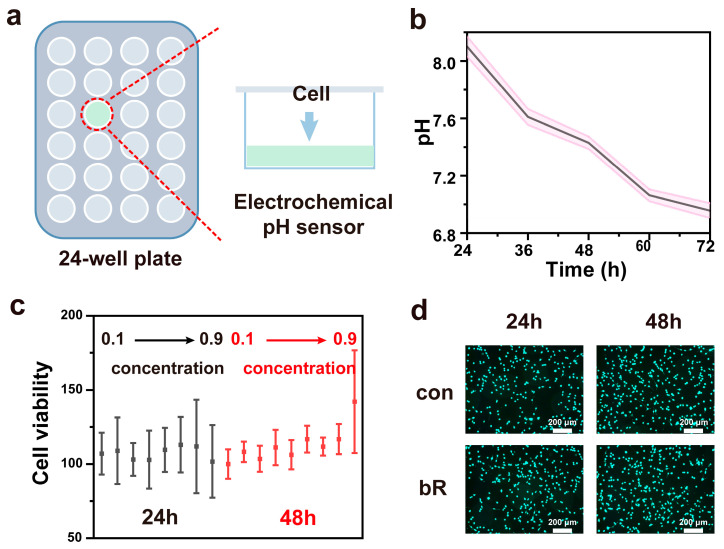
Medium pH measurements: (**a**) methods and workflow diagram for cell culture condition monitoring; (**b**) continuous pH measurement of B16-F10 cell culture (Red is the error band); (**c**) cell viability after co-incubated with a bR-based pH sensor with different concentrations; and (**d**) fluorescence images of the cells co-incubated with a bR-based pH sensor. (scale bar: 200 μm).

The integrated bR-based pH sensor features an integrated, flexible PET substrate that can be well incorporated into the bottom of the culture medium (Figure 4a). When measuring the pH, only xenon light needs to be applied to the cell culture medium. We monitored the pH of the culture medium over a 72 h period during cell growth, as exemplified in Figure 4b. To evaluate the stability of the device, the prepared bR-based electrode was stored in the environment for more than two months. Subsequently, its open-circuit potential drifted by less than 0.2 mV within a 7-day period (refer to Supplementary Materials Figure S2).

In addition to preventing silver ion leakage and extending the shelf life, the sterilization method in a cell culture environment further highlights the significance of integrated bR-pH sensors. Ethanol (70–80% *v*/*v*) is a commonly employed method for sterilizing laboratory equipment in biosafety settings. We disinfected the bR-based pH sensor by spraying it with a 75% *v*/*v* ethanol sterilizing solution. A comparison of open-circuit potentiometric measurements indicated that the pH sensor was not affected by the ethanol spray [25].

As illustrated in Figure 4c, the statistical charts depict cell viability after co-culturing cells with the bR-based pH sensor for 24 h and 48 h. Figure 4d presents the live/dead fluorescence images of the cells following 24 h and 48 h co-cultures with the bR-based pH sensor. It is evident that cell density undergoes a substantial increase as the incubation duration is prolonged. Specifically, after being co-cultured with the bR-based pH sensor at varying concentrations for 24 and 48 h, all experimental groups exhibited a cell viability exceeding 95%, thereby substantiating the remarkable biocompatibility of the pH sensor.

By incorporating bR-based pH sensors into cell culture systems like cell culture plates, non-invasive pH monitoring becomes feasible, enabling the tracking of pH alterations during cell growth. Such a monitoring approach exhibits not only biocompatibility but also offers the functionality to monitor cell growth cycles, ascertain media change intervals, and modulate cell growth conditions.

## 4. Discussion

### 4.1. Enhancement of Sensor Sensitivity and Stability for pH Monitoring

Exploration is underway to devise methods for chemically modifying or genetically engineering bR to augment its responsiveness to minute pH alterations. Specifically, site-directed mutagenesis is being employed to modify the crucial amino acid residues implicated in proton transfer, with the aim of scrutinizing the impact of such mutants on the pH sensing efficacy [26]. Furthermore, it is noteworthy that other factors could also induce color changes in bR; for instance, the electric field (i.e., the electrochromic or Stark effect) and temperature (i.e., thermochromism). The acidity and alkalinity induce color changes in bR, as well. The pH-induced structural changes in bR might be correlated to the surface charge asymmetry of bR film, as the cytoplasmic surface of bR film is more negative than its extracellular surface above a particular pH. A detailed study is being carried out to analyze the long-term stability of bR under diverse environmental circumstances, including temperature, humidity, and light intensity. The aim is to formulate a comprehensive stability model. Moreover, to counteract the degradation and denaturation of bR, two primary strategies are being pursued [27].

### 4.2. Integration of pH Sensor with Cell Culture Systems

Specialized sensor interfaces and fixation mechanisms are being designed and fabricated to accommodate different sizes and types of cell culture vessels, such as flasks, Petri dishes, and bioreactors. These custom-designed components will enable seamless and secure attachment of the sensors, ensuring that they do not disrupt essential cell culture procedures like cell seeding and medium replacement [28].

By comparing the physiological characteristics of cells cultured with and without the sensor, the biocompatibility and microenvironmental impact of the sensor can be quantified. If any adverse effects are detected, strategies such as material optimization, structural redesign, or the development of protective coating technologies will be pursued to minimize sensor–cell interactions and ensure accurate pH monitoring without compromising cell viability and normal growth [29].

## 5. Conclusions

An integrated electrochemical electrode tailored for pH monitoring has been devised. Its diminutive size and flexibility permit effortless integration into traditional cell culture plates, facilitating real-time pH surveillance. Notably, the pH sensing component of this electrode is a bio-based protein, conferring enhanced biocompatibility. The sensor demonstrates the capacity to concurrently measure the pH within physiologically pertinent ranges with commendable accuracy and precision, and it operates proficiently in both static and flowing media. The application of screen-printing technology affords more accurate regulation of the Ag/AgCl thickness and architecture. This leads to a partial reduction in the release of Ag ions and an overall enhancement in the stability, sterilizability, and biocompatibility of the entire system.

## Data Availability

All data is contained within the article.

## Supplementary Materials

The following supporting information can be downloaded at: https://www.mdpi.com/article/10.3390/ma18030478/s1, Figure S1: The image of the bR-based pH sensor; Figure S2: Stability of the bR-based pH sensor.

## References

[1] Qiu W., Lin X., Nagl S. (2024). In Situ Live Monitoring of Extracellular Acidosis near Cancer Cells Using Digital Microfluidics with an Integrated Optical pH Sensor Film. Anal. Chem..

[2] Lin J., Li X.-R., Zhao L.-Y., Li G.-P., Shen H.-Y., Li Y.-T., Ren T.-L. (2023). Review on Bacteriorhodopsin-Based Self-Powered Bio-Photoelectric Sensors. Mater. Sci. Semicond. Process..

[3] Yang Y., Gao X., Widdicombe B., Zhang X., Zielinski J.L., Cheng T., Gunatilaka A., Leung K.K., Plaxco K.W., Rajasekharan Unnithan R. (2024). Dual-Purpose Aptamer-Based Sensors for Real-Time, Multiplexable Monitoring of Metabolites in Cell Culture Media. ACS Nano.

[4] Xiao L., Li K., Liu B., Tu J., Li T., Li Y.-T., Zhang G.-J. (2023). A pH-Sensitive Field-Effect Transistor for Monitoring of Cancer Cell External Acid Environment. Talanta.

[5] Lv Y., Yang N., Li S., Lu S., Xiang Y. (2019). A Novel Light-Driven pH-Biosensor Based on Bacteriorhodopsin. Nano Energy.

[6] Lv Y., Liang D., Lu S., Aurbach D., Xiang Y. (2021). Unidirectional Electron Injection and Accelerated Proton Transport in Bacteriorhodopsin Based Bio-p-n Junctions. Biosens. Bioelectron..

[7] Mostafa H.I.A., Elfiki A.A. (2024). Bacteriorhodopsin of Purple Membrane Reverses Anisotropy Outside the pH Range of Proton Pumping Based on Logic Gate Realization. Sci. Rep..

[8] Proksch E. (2018). pH in Nature, Humans and Skin. J. Dermatol..

[9] Shigeta A., Otani Y., Miyasa R., Makino Y., Kawamura I., Okitsu T., Wada A., Naito A. (2022). Photoreaction Pathways of Bacteriorhodopsin and Its D96N Mutant as Revealed by in Situ Photoirradiation Solid-State NMR. Membranes.

[10] Mostafa H.I.A. (2023). Exploring Isotropic Tendency for the Blue Membrane Containing Wild-Type Bacteriorhodopsin. Biophys. Chem..

[11] Inoue K., Tsunoda S.P., Singh M., Tomida S., Hososhima S., Konno M., Nakamura R., Watanabe H., Bulzu P.-A., Banciu H.L. (2020). Schizorhodopsins: A Family of Rhodopsins from Asgard Archaea That Function as Light-Driven Inward H^+^ Pumps. Sci. Adv..

[12] Li Y., Huang A., Zhang T., Wen L., Shi Z., Shi L. (2022). A pH Monitoring Algorithm for Orifice Plate Culture Medium. Appl. Sci..

[13] Li Z., Zhang R., Xu F., Yang J., Zhou L., Mao H. (2023). A Cell State Monitoring System with Integrated In Situ Imaging and pH Detection. Sensors.

[14] Li H., Wang M., Qi G., Xia Y., Li C., Wang P., Sheves M., Jin Y. (2020). Oriented Bacteriorhodopsin/Polyaniline Hybrid Bio-Nanofilms as Photo-Assisted Electrodes for High Performance Supercapacitors. J. Mater. Chem. A.

[15] Maximychev A.V., Kholmansky A.S., Levin E.V., Rambidi N.G., Chamorovsky S.K., Kononenko A.A., Erokhin V.V., Checulaeva L.N. (1992). Oriented Purple Membrane Multilayers of Halobacteria Fabricated by Langmuir–Blodgett and Electrophoretic Sedimentation Techniques. Adv. Mater. Opt. Electron..

[16] Shin Y., Lee H.S., Hong Y.J., Sunwoo S.-H., Park O.K., Choi S.H., Kim D.-H., Lee S. (2024). Low-Impedance Tissue-Device Interface Using Homogeneously Conductive Hydrogels Chemically Bonded to Stretchable Bioelectronics. Sci. Adv..

[17] Xu Z., Qiao X., Tao R., Li Y., Zhao S., Cai Y., Luo X. (2023). A Wearable Sensor Based on Multifunctional Conductive Hydrogel for Simultaneous Accurate pH and Tyrosine Monitoring in Sweat. Biosens. Bioelectron..

[18] Shan Y., Xu L., Cui X., Wang E., Jiang F., Li J., Ouyang H., Yin T., Feng H., Luo D. (2024). A Responsive Cascade Drug Delivery Scaffold Adapted to the Therapeutic Time Window for Peripheral Nerve Injury Repair. Mater. Horiz..

[19] Zhao Z., Wang P., Xu X., Sheves M., Jin Y. (2015). Bacteriorhodopsin/Ag Nanoparticle-Based Hybrid Nano-Bio Electrocatalyst for Efficient and Robust H _2_ Evolution from Water. J. Am. Chem. Soc..

[20] Jin Y., Honig T., Ron I., Friedman N., Sheves M., Cahen D. (2008). Bacteriorhodopsin as an Electronic Conduction Medium for Biomolecular Electronics. Chem. Soc. Rev..

[21] Sheves M., Albeck A., Friedman N., Ottolenghi M. (1986). Controlling the pKa of the Bacteriorhodopsin Schiff Base by Use of Artificial Retinal Analogues. Proc. Natl. Acad. Sci. USA.

[22] Tahara S., Kuramochi H., Takeuchi S., Tahara T. (2019). Protein Dynamics Preceding Photoisomerization of the Retinal Chromophore in Bacteriorhodopsin Revealed by Deep-UV Femtosecond Stimulated Raman Spectroscopy. J. Phys. Chem. Lett..

[23] Rahmatnejad V., Tolosa M., Ge X., Rao G. (2024). Completely Noninvasive Multi-Analyte Monitoring System for Cell Culture Processes. Biotechnol. Lett..

[24] Li Y., Wu M., Zhao D., Wei Z., Zhong W., Wang X., Liang Z., Li Z. (2015). Electroporation on Microchips: The Harmful Effects of pH Changes and Scaling Down. Sci. Rep..

[25] Lee H.E. (2021). Novel Bio-Optoelectronics Enabled by Flexible Micro Light-Emitting Diodes. Electronics.

[26] Xu B., Li B., Zhang J., Tong J., Liu Y. (2024). Unveiling the Effect of Ti Micro-Alloying on the Microstructure and Corrosion Resistance of the GH3536 Alloy Processed by Laser Metal Deposition in a Simulated Environment for PEMFCs. Materials.

[27] Qu X., Yang Z., Cheng J., Li Z., Ji L. (2023). Development and Application of Nanogenerators in Humanoid Robotics. Nano Trends.

[28] Drymiskianaki A., Viskadourakis Z., Kenanakis G. (2024). Hybrid Microwave/Solar Energy Harvesting System Using 3D-Printed Metasurfaces. Materials.

[29] Gao Y., Li H., Chao S., Wang Y., Hou L., Bai T., Bai J., Man X., Cui Z., Wang N. (2024). Zebra-Patterned Stretchable Helical Yarn for Triboelectric Self-Powered Multifunctional Sensing. ACS Nano.

