# Supplementary material for: Bacteriorhodopsin-Based pH Sensor for Cell Culture Condition Regulation
Source: Materials (Basel). 2025 Jan 21;18(3):478. doi: 10.3390/ma18030478 (PMC11818522; doi:10.3390/ma18030478)
Supplement: Supplementary file 1 [file materials-18-00478-s001.zip › materials-3395564-supplementary.pdf]

materials

Article

Bacteriorhodopsin-Based pH Sensor for Cell Culture Condition Regulation

Jiayin Huang [1,2,3,4], Shiwang Xie [2,5], Haoqi Fan [2,6], Chen Song [2,7], Qiang Zheng [1,3,4*], Dan Luo [2,*], Zhu Zeng [1,3,*], Zhou Li [2,5,*] and Yujia Lv [2,*]

1 Key Laboratory of Infectious Immune and Antibody Engineering of Guizhou Province, Engineering Research Center of Cellular Immunotherapy of Guizhou Province, School of Biology and Engineering, Guizhou Medical University, Guiyang 561113, China; huangjiayin@binn.cas.cn
2 Beijing Institute of Nanoenergy and Nanosystems, Chinese Academy of Sciences, Beijing 101400, China; xieshiwang@binn.cas.cn (S.X.); fanhaoqi@binn.cas.cn (H.F.); songchen@binn.cas.cn (C.S.)
3 Immune Cells and Antibody Engineering Research Center of Guizhou Province, Key Laboratory of Biology and Medical Engineering, Guizhou Medical University, Guiyang 561113, China
4 Engineering Research Center of Intelligent Materials and Advanced Medical Devices, School of Biology and Engineering, Guizhou Medical University, Guiyang 561113, China
5 School of Nanoscience and Technology, University of Chinese Academy of Sciences, Beijing 100049, China
6 Key Laboratory of Urban Stormwater System and Water Environment, Ministry of Education, Beijing University of Civil Engineering and Architecture, Beijing 100044, China
7 Faculty of Electronic Information and Automation, Tianjin University of Science and Technology, Tianjin 300457, China
* Correspondence: zhengqiang@gmc.edu.cn (Q.Z.); luodan@binn.cas.cn (D.L.); zengzhu@gmc.edu.cn (Z.Z.); zli@binn.cas.cn (Z.L.); lvyujia@binn.cas.cn (Y.L.)

Academic Editor: Keisuke Nakano

Received: 12 December 2024
Revised: 9 January 2025
Accepted: 11 January 2025
Published: 21 January 2025

Citation: Huang, J.; Xie, S.; Fan, H.; Song, C.; Zheng, Q.; Luo, D.; Zeng, Z.; Li, Z.; Lv, Y. Bacteriorhodopsin-Based pH Sensor for Cell Culture Condition Regulation. *Materials* **2025**, *18*, x. https://doi.org/10.3390/xxxxx

Copyright: © 2025 by the authors. Submitted for possible open access publication under the terms and conditions of the Creative Commons Attribution (CC BY) license (https://creativecommons.org/licenses/by/4.0/).

Abstract: In cell culture research and biotechnology, precise pH monitoring is crucial for maintaining cellular health and ensuring reliable experimental outcomes. Traditional pH measurement methods, such as glass electrodes and chemical indicators, are often limited by issues such as fragility, calibration requirements, and potential cytotoxicity. This study presents a novel pH sensor based on bacteriorhodopsin (bR), a light-sensitive protein that undergoes conformational changes in response to pH fluctuations, generating a measurable photoelectric signal. The integrated bR-based electrochemical electrode in a flexible pH biosensor is demonstrated, with measurements spanning the physiological pH range of 6.0–8.5. The sensor shows a high correlation (R^2 = 0.977) between photo-generated current signals and pH, indicating robust performance for real-time, non-invasive pH monitoring. The biocompatibility and non-invasive nature of this sensor make it particularly suitable for continuous monitoring in cell culture environments. The sensor's practical application is validated by its integration into cell well plates for tracking the pH changes during cell growth, providing valuable insights into metabolic processes and growth conditions. In the future, efforts will focus on enhancing sensor sensitivity, stability, and integration with multi-parameter monitoring systems for more comprehensive cell culture analysis.

Keywords: bacteriorhodopsin; pH sensor; cell culture

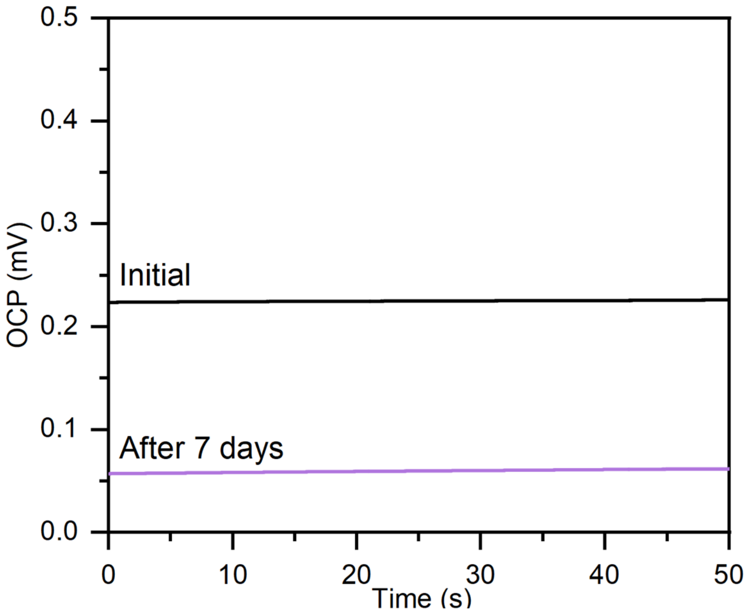

Figure S1. Stability of the bR-based pH sensor.

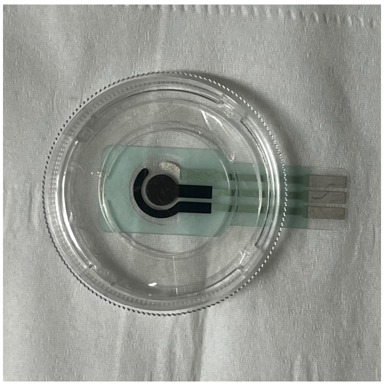

Figure S2. The image of the bR-based pH sensor.
